# Is Upregulation of Aquaporin 4-M1 Isoform Responsible for the Loss of Typical Orthogonal Arrays of Particles in Astrocytomas?

**DOI:** 10.3390/ijms17081230

**Published:** 2016-07-29

**Authors:** Petra Fallier-Becker, Maike Nieser, Ulrike Wenzel, Rainer Ritz, Susan Noell

**Affiliations:** 1 Institute of Pathology and Neuropathology, University Hospital of Tuebingen, Liebermeisterstr. 8, 72076 Tuebingen, Germany; petra.fallier-becker@med.uni-tuebingen.de (P.F.-B.); ulrike.wenzel@med.uni-tuebingen.de (U.W.); 2 Department of Neurosurgery, Schwarzwald-Baar Klinikum, Klinikstr. 11, 78052 Villingen-Schwenningen, Germany; rainer_ritz@hotmail.com; 3 Department of Neurosurgery, University Hospital of Tuebingen, Hoppe-Seyler-Str. 3, 72076 Tuebingen, Germany; dr.susan.noell@googlemail.com

**Keywords:** low-grade astrocytoma, high-grade astrocytoma, malignancy, aquaporin

## Abstract

The astrocytic endfoot membranes of the healthy blood-brain barrier—contacting the capillary—are covered with a large number of the water channel aquaporin 4 (AQP4). They form orthogonal arrays of particles (OAPs), which consist of AQP4 isoform M1 and M23. Under pathologic conditions, AQP4 is distributed over the whole cell and no or only small OAPs are found. From cell culture experiments, it is known that cells transfected only with AQP4-M1 do not form OAPs or only small ones. We hypothesized that in astrocytomas the situation may be comparable to the in vitro experiments expecting an upregulation of AQP4-M1. Quantitative Real-time PCR (qRT-PCR) of different graded astrocytomas revealed an upregulation of both isoforms AQP4 M1 and M23 in all astrocytomas investigated. In freeze fracture replicas of low-grade malignancy astrocytomas, more OAPs than in high-grade malignancy astrocytomas were found. In vitro, cultured glioma cells did not express AQP4, whereas healthy astrocytes revealed a slight upregulation of both isoforms and only a few OAPs in freeze fracture analysis. Taken together, we found a correlation between the decrease of OAPs and increasing grade of malignancy of astrocytomas but this was not consistent with an upregulation of AQP4-M1 in relation to AQP4 M23.

## 1. Introduction

Aquaporin 4 (AQP4) is the main water channel in the mammalian brain. It is morphologically assembled in square arrays called orthogonal arrays of particles (OAPs) [1,2,3] localized in superficial and perivascular astrocytic end foot membranes at the blood-brain barrier (BBB). In parenchymal membranes where the astrocyte loses contact to the basal lamina, only a small number of OAPs is found, indicating the polarization of astrocytes [4]. This polarization disappears in astrocytomas. Here, AQP4 protein is strongly upregulated compared to healthy brain tissue as was shown by Saadoun et al. by immunohistological stainings [5]. However, Noell et al. [6] reported that freeze fracture replicas of glioblastomas revealed no or only small OAPs. Western blot analyses of AQP4 expressing cells and tissues revealed two bands for AQP4 that refer to two different AQP4 isoforms: AQP4-M23 and AQP4-M1 with M1 being 22 amino acids (AAs) longer than M23 [7,8]. It is known from in vitro transfection experiments that cells transfected with AQP4-M23-isoform form large lattices of OAPs whereas in M1-transfected cells no or only small OAPs were observed [9,10]. Consequently, we hypothesized that in astrocytomas, where no typical OAPs have been found in freeze fracture replicas, AQP4-M1 might be upregulated in relation to AQP4-M23. In this work we examined tissues from patients with low-grade to high-grade astrocytomas (World Health Organization (WHO) grade II to IV) asking the question whether or not the AQP4-M1 mRNA is upregulated compared to AQP4-M23 mRNA. Furthermore, we performed freeze fracture analysis of these glioma tissues in order to find out if there is a correlation between the number of OAPs and the grade of malignancy. In addition, cultured glioma cells were analyzed in the same way and compared to normal murine astrocytes in vitro.

## 2. Results

### 2.1. Determination of Orthogonal Arrays of Particles (OAPs) from Low-Grade to High-Grade Malignancies

**Figure 1:**
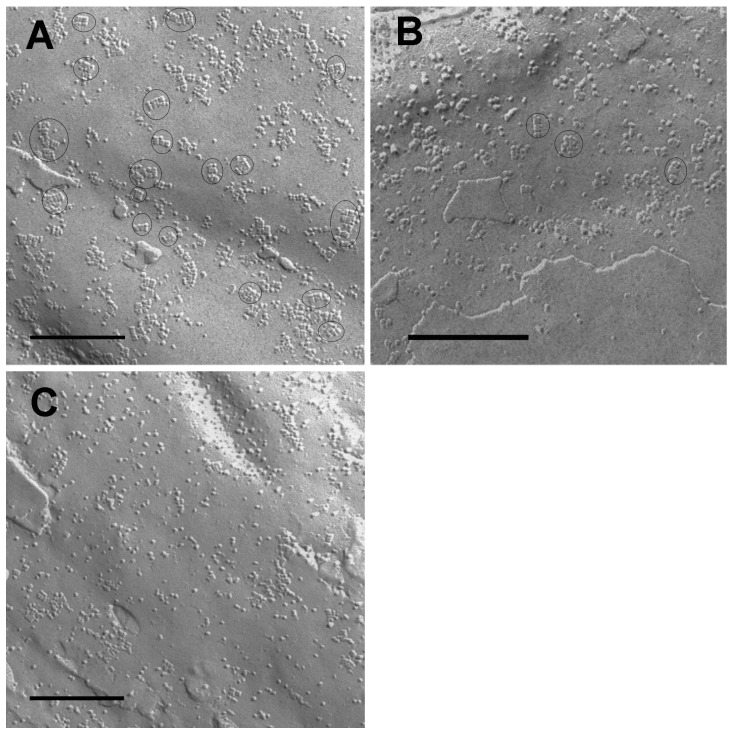
Freeze fracture replicas of low-grade to high-grade astrocytomas. (**A**) Astrocytoma World Health Organization (WHO) grade II showing orthogonal arrays of particles (OAPs) (encircled); (**B**) Astrocytoma WHO grade III showing less OAPs than in A (encircled); (**C**) IDH1 (isocitrate dehydrogenase 1 (NADP(+))-negative glioblastoma: No typical OAPs are found. Bar: 250 nm.

Using freeze fracture techniques, we analyzed replicas of human astrocytomas from low-grade to high-grade malignancy in order to find out whether or not there is a relationship between the number of typical OAPs and the grade of malignancy of these astrocytomas. Figure 1 shows the freeze fracture replicas of the examined astrocytoma tissues: low-grade astrocytomas (WHO grade II; Figure 1A) revealed membranes with OAPs. The astrocytoma WHO grade III (Figure 1B) showed a few OAPs. IDH1 (isocitrate dehydrogenase 1 (NADP(+))-negative (non-mutated) glioblastomas (Figure 1C) formed only few and sometimes misshapen OAPs or membranes lacking any OAPs.

**Figure 2:**
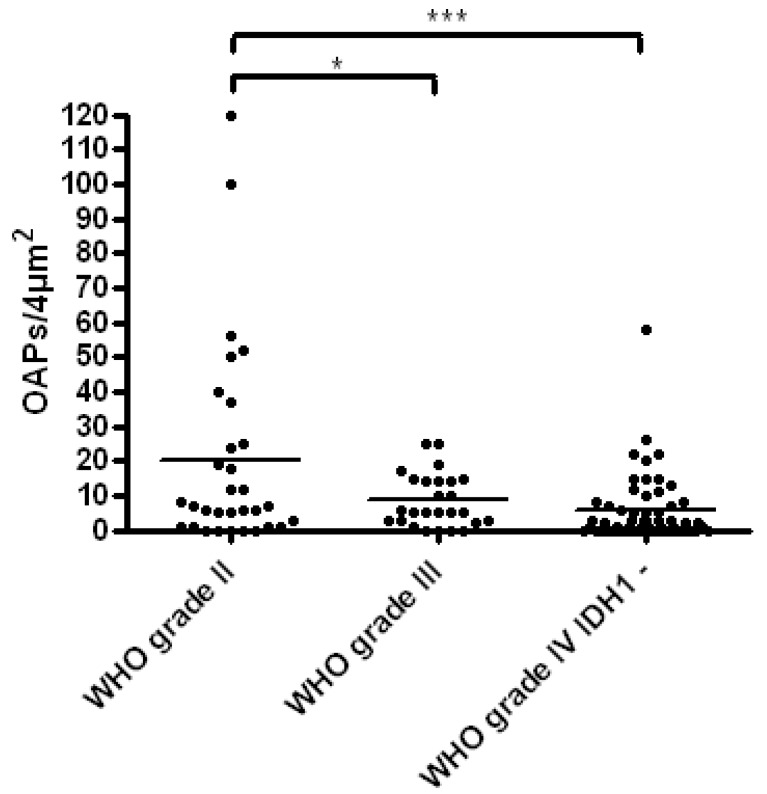
Number of OAPs counted per 4 µm^2^ in astrocytoma WHO grade II, III and IV IDH1-negative glioblastoma. The number of OAPs in astrocytoma WHO grade II is significant higher than in astrocytoma WHO grade III (*p* = 0.028) and glioblastoma (*p* = 0.0006). * *p* ≤ 0.05, *** *p* ≤ 0.001.

Quantification was performed by counting OAPs of all astrocytoma tissues per 4 µm^2^. The results are shown in Figure 2 indicating that astrocytomas WHO graded II harbor significantly more OAPs than astrocytomas WHO grade III (*p* = 0.028) and glioblastomas (WHO graded IV; *p* < 0.001). These results point out that there is a correlation between the formation of OAPs and the grade of malignancy in glial tumors of the human brain.

### 2.2. Determination of Aquaporin 4 (AQP4)-M1 and -M23 mRNA in Low-Grade and High-Grade Astrocytomas

**Figure 3:**
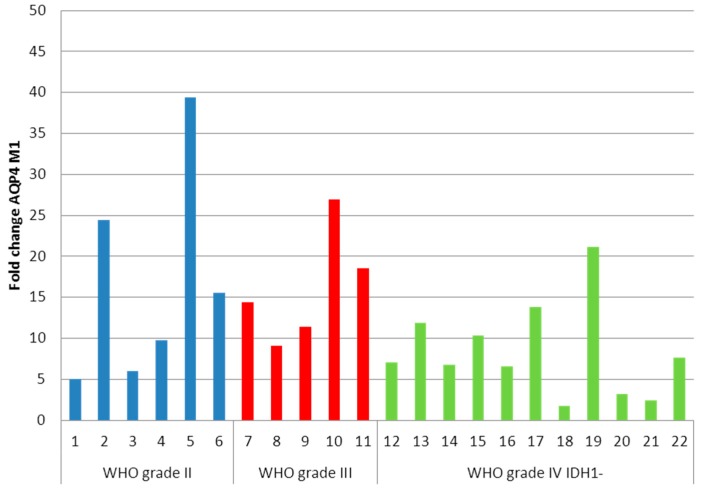
Aquaporin 4 (AQP4)-M1 fold changes of astrocytomas. The average fold change is 12.41. No significant differences between the WHO grade groups were detected.

We know from cell culture experiments that cells transfected with AQP4-M23 isoform are forming large OAP lattices whereas M1-transfected cells show no or only small OAPs [6,7]. Consequently, we performed qRT-PCR to investigate the expression of the two AQP4 isoforms in our patients’ tumor samples. AQP4-M1 expression was upregulated in all astrocytomas, without any correlation to the grade of malignancy (Figure 3).

**Figure 4:**
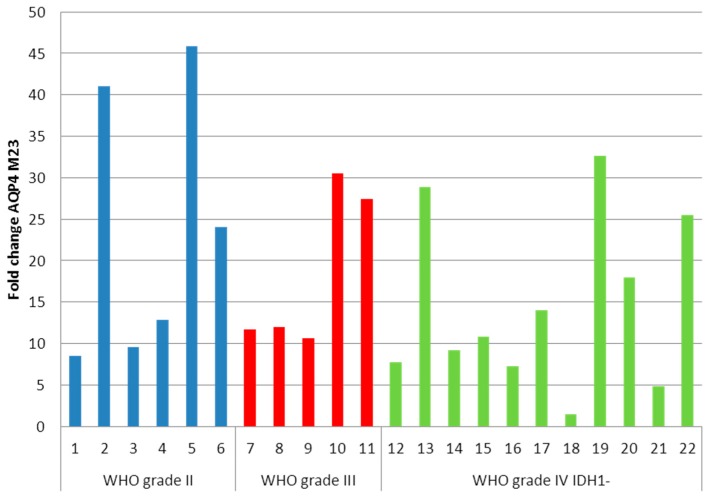
AQP4-M23 fold changes of astrocytomas. The average fold change is 17.93. No significant differences between the WHO grade groups were detected.

The average fold change of AQP4-M1 was 12.41 compared to a normal brain. Surprisingly, the average fold change of AQP4-M23 was 17.93 (Figure 4), indicating that AQP4-M23 was even more upregulated than AQP4-M1.

The *M23*/*M1* ratios ranged between 0.81 and 5.58, with an average ratio of 1.64, indicating that most gliomas express the M23 isoform over 1.5-fold more than M1. When comparing the different astrocytoma grades, astrocytoma II and III depict ratios of 1.5 and 1.14, respectively. The average ratio for astrocytomas IV IDH1- is 1.94. It seems that *AQP4-M23*/*M1* ratio is higher in the more malignant IDH1-negative glioblastomas than in the IDH1-positive astrocytomas.

### 2.3. Determination of AQP4-M1 and -M23 mRNA in Cultures of Normal Astrocytes and Glioma Cells

**Figure 5:**
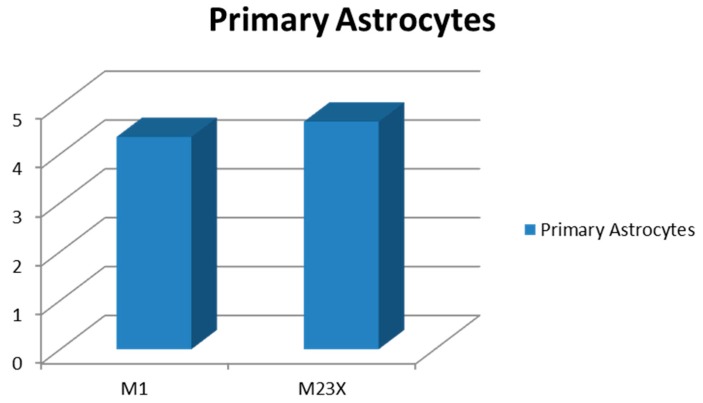
AQP4 M1 and M23X fold changes of primary astrocytes. Both isoforms depict 4–5-fold expression compared to the normal mouse brain.

**Figure 6:**
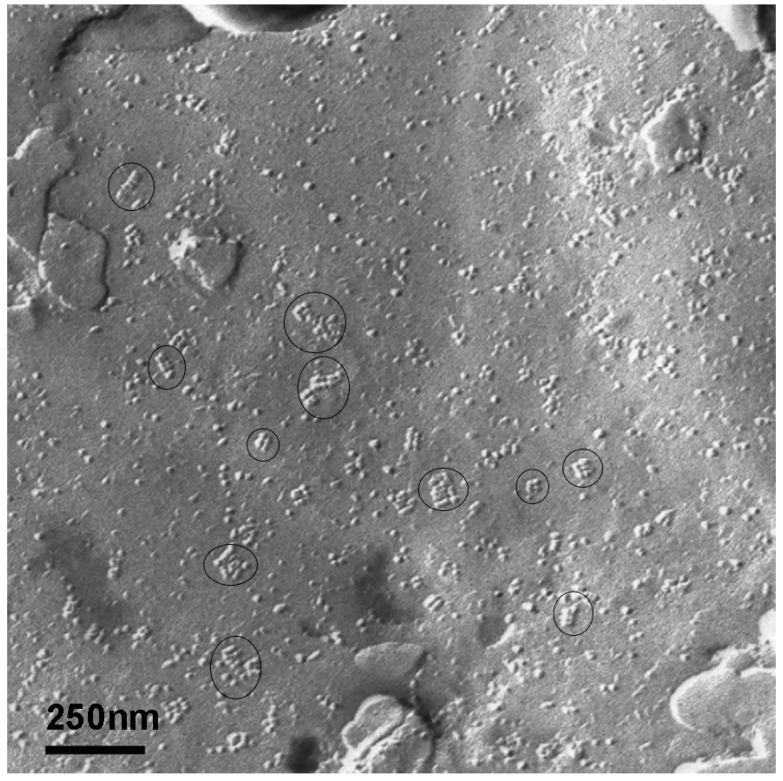
Freeze fracture replica of a healthy murine astrocyte in culture showing OAPs encircled.

Cell cultures of murine astrocytes from healthy brain and human glioma cells (TuGlio 25 and U373) were processed for qRT-PCR to compare their expression of AQP4 isoforms to the patients’ tumor tissue. Cultured mouse brain astrocytes revealed a slight upregulation of both AQP4-M1 (fold change 4.35) and AQP4-M23X (fold change 4.66, Figure 5) compared to a normal brain. Freeze fracture replicas of mouse astrocytes showed several small sized OAPs (Figure 6).

In U373 and TuGlio25 tumor cells, however, both isoforms were expressed at a very low level (average AQP4-M1 crossing point (CP) values of 32.04 and 34.54 for TuGlio25 and U373, and average AQP4-M23 CP values of 35 and 32.89, respectively) indicating that AQP4 is not expressed in these human tumor cell cultures. Accordingly, no OAPs were formed.

## 3. Discussion

In this work, we furnished proof that the number of OAPs in human astrocytomas decreased from low-grade to high-grade malignancy although the amount of AQP4 protein was increasing [11]. We hypothesized that there might be a connection to the in vitro transfection results of Furman et al. [9] who found no or only small OAPs in AQP4-M1 transfected cell cultures. From these findings, we concluded that astrocytomas revealing no typical OAPs should express more AQP4-M1 than AQP4-M23 compared to the normal brain. However, not only AQP4-M1 but alsoAQP4-M23 was upregulated, even to a slightly higher degree than AQP4-M1. Astrocytomas WHO graded II and III depict average *M23*/*M1* ratios of 1.5 and 1.14, whereas the average *M23*/*M1* ratio of glioblastomas is elevated to 1.94. In the healthy brain, however, the *M23*/*M1* is at least 3 [9]. In addition, Jin et al. report that low *M23*/*M1* ratios yield small OAPs, whereas high *M23*/*M1* ratios lead to large OAPs [12]. Our results show that in low-grade and high-grade astrocytomas more AQP4-M23 isoform than AQP4-M1 was expressed despite the decreasing number of OAPs compared to a normal brain. Investigating AQP4-M1 and -M23 mRNA expression of cell cultures of primary tumor cells (TuGlio25) and a tumor cell line (U373) revealed very low AQP4 mRNA expression levels. These results are consistent with the immunohistological investigations of C6 rat glioma cell cultures, which yielded no positive staining for AQP4 [12]. In contrast, in cultures of healthy murine astrocytes, AQP4–M23 and –M1 mRNA were expressed. Here we found the *M23*/*M1* ratio of 1.07 which is comparable to *M23*/*M1* ratios of astrocytomas WHO grade III. In addition, the formation of OAPs in healthy astrocyte cultures was also comparable to the OAPs found in astrocytomas WHO grade II.

Taken together we conclude that loss of OAPs in astrocytomas depends on other factors than the upregulation of AQP4-M1 alone. Former western blot investigations of aquaporin 4 in glioblastoma tissues [6] and rat brain gliomas [13] always yielded the same results: the AQP4-M23 bands were thicker than the M1 bands independent of the formation of OAPs in the appropriate membranes. It seems that the formation of OAPs underlies other mechanisms than change of the *M23*/*M1* ratio. As reported previously, the microenvironment of brain tumors differs from that of normal brains [6]. Our previous findings revealing a correlation between the presence of agrin in the extracellular matrix and the formation of OAPs in glioblastoma and agrin knock-out mice [6,14] suggest that this is also the case in astrocytomas and might be the reason for the decreasing number of OAPs with increasing malignancy of the astrocytomas. In addition to agrin, dystroglycan is also fundamental in forming OAPs. It links agrin in the extracellular matrix via utrophin to alpha synthrophin and to AQP4 [15]. Although investigation of subependymomas reveals also no expression of agrin and dystroglycan and hence no formation of OAPs, these benign tumors are not comparable to the infiltrating astrocytomas [16]. In addition, the activity of matrixmetalloproteinases MMP2, 9, and 3 play a pivotal role in this scenario: if agrin is cleaved by MMP3 [17]—such as in glioblastoma—no typical OAPs are found and if MMP2 and 9 cleave dystroglycan [18]—such as in glioblastoma—no OAPs are formed as well. All these in vivo investigations suggest that the microenvironment in the brain is more important for the formation of OAPs than the *M23*/*M1* relation (Table 1). Furthermore, in vitro experiments showed that murine astrocyte cultures form more OAPs when growing on agrin-coated surfaces or in agrin-containing culture medium [19,20].

It still remains unclear why AQP4 form OAPs and what their role might be. It has been suggested that AQP4 water channels might have a better adhesion to the neighbored membrane if assembled in square arrays [8,21]; others propose a more effective water exchange when AQP4-molecules are packed in OAPs [22]. The isoform AQP4-M23 is suggested to be the faster water channel because it works more effectively in OAPs [23].

## 4. Materials and Methods

### 4.1. Tissue

Tumor tissues from 22 patients with astrocytoma graded WHO II, III, and IV (Table 2) were analyzed using freeze fracture techniques and qRT-PCR. The ethics committee of the medical faculty of the Eberhard-Karls University of Tuebingen approved the project and patients’ consent procedures. The ethics committee waived the need for consent (project no. 663/2013BO2). All patients were treated in the Department of Neurosurgery Tuebingen between 2013 and 2014 (Table 2).

### 4.2. Cell Culture

Human primary glioma cells were isolated enzymatically from a patient’s glioblastoma. Briefly, resected surgical material was transported under sterile conditions in a 4-(2-hydroxyethyl)-1-piperazineethanesulfonic acid (HEPEs) buffered medium (Thermo Fisher Scientific, Waltham, MA, USA) and cells were isolated using trypsin (Sigma, Taufkirchen, Germany). After centrifugation, culture medium was added to the cell pellet and cell suspension was pipetted on a cell strainer (70 µm) to get single cells that were seeded in culture flasks and cultured in a humidified incubator at 37 °C and 5% CO_2_ under standard conditions with Dulbecco's modified Eagle’s medium (DMEM) (Thermo Fisher Scientific, Waltham, MA, USA) supplemented with 10% fetal calf serum (Thermo Fisher Scientific, Waltham, MA, USA), 1% penicillin and streptomycin (Sigma-Aldrich, St. Louis, MO, USA). Culture medium was changed every two days.

Ulrike Naumann, Hertie Institute of Clinical Research, Tuebingen, Germany, kindly provided U373 cells. They were cultured as described above.

Murine cortex astrocytes were isolated from 5 day old mice as described above and cultured under standard conditions [24,25]. When cells reached confluence they were processed for qRT-PCR and freeze fracture analysis.

### 4.3. Freeze Fracture Technique and Determination of OAP Densities

Tissues and cells were processed for freeze fracture analysis as described before [6].

### 4.4. RNA Extraction

Cryo tumor specimens of the above-mentioned astrocytomas were gathered from the surgical pathology files of the Department of Neuropathology of the Institute of Pathology of Tuebingen. RNA of the samples and the cell cultures was extracted manually with the RNeasy Mini Kit (Qiagen, Hilden, Germany), following the manufacturer’s instructions.

RNA quantification was performed with the NanoDrop ND-2000 (Thermo Fisher Scientific) spectrophotometer.

Human Brain, Cerebral Cortex Total RNA, and Mouse Brain Total RNA (Clontech, Mountain View, CA, USA) served as healthy controls for qRT-PCR.

### 4.5. cDNA Synthesis

Intron-spanning oligonucleotides for human *HPRT1* (housekeeping gene), *AQP4 M1*, and *AQP4 M23*, as well as murine *Hprt1*, *Aqp4 M1*, and *Aqp4 M23X* [26] were designed using the program Primer3Plus (http://primer3plus.com/cgi-bin/dev/primer3plus.cgi). In Table 3 and Table 4 the primer sequences and the product sizes are shown. Primer-BLAST (http://www.ncbi.nlm.nih.gov/tools/primer-blast/) was used to check for the specificity of the primers.

### 4.6. Quantitative Real Time-PCR (qRT-PCR)

Total RNA (1 μg) was reversely transcribed using the High-Capacity cDNA RT Kit (Thermo Fisher Scientific) in combination with RNA Inhibitor (Thermo Fisher Scientific). The qRT-PCR mix included 10 μL Real-Time SYBR Green PCR master mix, 1 μL (20 ng) diluted reverse transcription product, 2 μL each of Primer (Table 2 and Table 3) and 7 μL DNase/RNase free water. The PCR was carried out under following conditions: 95 °C for 15 min followed by 40 cycles of 94 °C for 15 s, 55 °C for 30 s and 70 °C for 30 s in a LightCycler 480 II (Roche, Basel, Switzerland). Normal brain controls as well as a negative water control were included in every run. All samples were run in triplicates.

By running the PCR for 40 cycles, the maximal crossing point (CP) value that could be achieved by the analysis was 35. The CP value is the number of PCR cycles at which a constant fluorescence level is achieved. CP values between 15 and 25 indicate strong expression, 25–30 medium expression, and >30 weak expression.

To assess the specificity of the amplified PCR product a melting curve analysis was carried out.

### 4.7. Data Analysis and Statistics

The average raw CP values (median of triplicates) were imported into Microsoft Excel (Version, Microsoft Corp., Seattle, WA, USA). The AQP4-M1 and -M23 expression were analyzed using the comparative ΔCP method, where ΔCP = CP candidate target − CP reference RNA. HPRT1 was used as endogenous reference gene for the analysis. ΔΔCP = ΔCP sample − ΔCP normal brain control, relative expression = 2^−ΔΔCP^, fold change: if relative expression >1 then fold change ≙ relative expression, if relative expression <1 then fold change = −1/relative expression.

*M23*/*M1* ratios were determined by simply dividing the M23 fold changes through the M1 fold changes. For the evaluation of the OAP densities and the AQP4-M1 and -M23 fold changes of the astrocytomas two-tailed unpaired *t*-tests were performed using GraphPad software (Version 4, San Diego, CA, USA). The scatter plot for the OAPs was also achieved by using this software.

## 5. Conclusions

Taken together we were able to demonstrate a significant correlation between the decreasing number of OAPs and the grade of malignancy in human astrocytomas. Furthermore, we found an upregulation of AQP4-M1 as well as an upregulation of -M23 mRNA in all astrocytomas but the *M23*/*M1* ratio differed from 1.14 to 1.5 in low-grade astrocytomas to 1.94 in glioblastomas. These results demand further investigations to elucidate the question whether or not they will yield an impact on the development of new therapeutics.

## Figures and Tables

**Table 1:** Comparison of typical features between healthy brain and glioblastoma.

Protein Expression/Protein Complex	Healthy Brain	Glioblastoma
Agrin	+	−
Dystroglycan	+	−
Active MMP3	−	+
Active MMP2/9	−	+
OAPs	+	−

**Table 2:** World Health Organization (WHO) grading and IDH1 (isocitrate dehydrogenase 1 (NADP(+)) status of patients.

Patient Number	Astrocytoma WHO Grade	IDH1
1	II	IDH1 (R132H) mutation (IDH1+)
2	II	IDH1 R132H) mutation (IDH1+)
3	II	IDH1 (R132H) mutation (IDH1+)
4	II	IDH1 (R132H) mutation (IDH1+)
5	II	IDH1 (R132H) mutation (IDH1+)
6	II	IDH1 (R132H) mutation (IDH1+)
7	III	IDH1 (R132H) mutation (IDH1+)
8	III	IDH1 (R132H) mutation (IDH1+)
9	III	IDH1 (R132H) mutation (IDH1+)
10	III	IDH1 (R132H) mutation (IDH1+)
11	III	IDH1 (R132H) mutation (IDH1+)
12	IV	IDH1 wildtype (IDH1−)
13	IV	IDH1 wildtype (IDH1−)
14	IV	IDH1 wildtype (IDH1−)
15	IV	IDH1 wildtype (IDH1−)
16	IV	IDH1 wildtype (IDH1−)
17	IV	IDH1 wildtype (IDH1−)
18	IV	IDH1 wildtype (IDH1−)
19	IV	IDH1 wildtype (IDH1−)
20	IV	IDH1 wildtype (IDH1−)
21	IV	IDH1 wildtype (IDH1−)
22	IV	IDH1 wildtype (IDH1−)

**Table 3:** Primer (*Homo sapiens*).

Primer	Sequence	Product Size
HPRT1 Ex6 for HPRT1 Ex7 rev	TGACACTGGCAAAACAATGC TTCGTGGGGTCCTTTTCACC	101 bp
AQP4 M1 Ex1 for AQP4 M1 Ex2 rev	GGGGAAGGCATGAGTGACAG AAAGCTTGAGTCCAGACCCC	110 bp
AQP4 M23 Ex1 for AQP4 M23 Ex2 rev	TCTCTTTTCAGTAAGTGTGGACCT CATGGCCAGAAATTCCGCTG	114 bp

**Table 4:** Primer (*Mus musculus*).

Primer	Sequence	Product Size
HPRT Ex6 Mus for HPRT Ex7 Mus rev	CAAACTTTGCTTTCCCTGGT GGCCTGTATCCAACACTTCG	91 bp
AQP4 M1 Mus Ex1 for AQP4 M1 Mus Ex2 rev	AGGGAAGGCATGAGTGACAG GACTCCTTTGAAAGCCACCA	96 bp
AQP4 M23X Mus for AQP4 M23X Mus rev	TATGGTTCACGGGTTTGGAT CCCTTTGTCACCTGCTCATT	139 bp
